# Digital Technology Ownership, Usage, and Factors Predicting Downloading Health Apps Among Caucasian, Filipino, Korean, and Latino Americans: The Digital Link to Health Survey

**DOI:** 10.2196/mhealth.3710

**Published:** 2014-10-22

**Authors:** Melinda S Bender, JiWon Choi, Shoshana Arai, Steven M Paul, Prisila Gonzalez, Yoshimi Fukuoka

**Affiliations:** ^1^University of California San FranciscoInstitute for Health & AgingDepartment of Social and Behavioral SciencesSan Francisco, CAUnited States; ^2^University of California San FranciscoSchool of Nursing Office of ResearchSan Francisco, CAUnited States; ^3^University of California San FranciscoFamily Health Care NursingSan Francisco, CAUnited States

**Keywords:** digital technology, mobile health apps, mHealth, Latinos, Filipinos, Koreans, cross-sectional survey

## Abstract

**Background:**

Interventions using mobile health (mHealth) apps have been effective in promoting healthy lifestyle behavior change and hold promise in improving health outcomes to thereby reduce health disparities among diverse racial/ethnic populations, particularly Latino and Asian American subgroups (Filipinos and Koreans) at high risk for diabetes and cardiovascular disease. Latinos and Asian Americans are avid digital technology owners and users. However, limited datasets exist regarding digital technology ownership and use, especially among specific racial/ethnic subgroups. Such information is needed to inform development of culturally tailored mHealth tools for use with lifestyle interventions promoting healthy behaviors for these at-risk racial/ethnic populations.

**Objective:**

The intent of the study was to examine (1) digital technology ownership and usage, and (2) factors predicting downloading health apps for Caucasian, Filipino, Korean, and Latino American subgroups.

**Methods:**

A cross-sectional survey conducted in August 2013 through December 2013 recruited 904 participants (Caucasians n=172, Filipinos n=250, Koreans n=234, and Latinos n=248), age >18 years, from California community events, clinics, churches, and online. English, Spanish, and Korean surveys were administered via paper or online. Descriptive statistics characterized the sociodemographics and digital technology ownership/usage of the 904 participants. Differences among groups in categorical variables were examined using chi-square statistics. Logistic regression was used to determine factors predicting downloading health apps.

**Results:**

Overall, mean age was 44 years (SD 16.1), with 64.3% (581/904) female. Only 44.7% (404/904) of all participants reported English as their primary language (Caucasian 98.3%, 169/172; Filipino 67.6%, 169/250; Korean 9.4%, 22/234, and Latino 17.7%, 44/248. Overall, mobile phone ownership was 92.8% (839/904). Compared to all groups, Koreans were more likely to own a mobile phone (82.8%, 194/234), computer (91.4%, 214/234), or tablet (55.2%, 129/234), whereas Latinos (67.5%, 167/248; 65.3%, 162/248; 24.4%, 61/248, respectively) were least likely. Internet access via mobile phones (90.5%, 818/904) was higher than computers (78.6%, 711/904). Odds of downloading health apps increased with college (OR 2.62, 95% CI 1.44-4.80) or graduate school (OR 2.93, 95% CI 1.43-6.00) compared to some high school; and family history of heart attack (OR 2.02, 95% CI 1.16-3.51). Odds of downloading health apps were reduced with: race/ethnicity, Latino (OR 0.37, 95% CI 0.20-0.69), and Korean (OR 0.52, 95% CI 0.31-0.88) compared to Caucasians; increasing age (OR 0.96, 95% CI 0.95-0.97); and completing paper surveys (OR 0.50, 95% CI 0.34-0.75).

**Conclusions:**

This survey study uniquely targeted specific racial/ethnic subgroups. Results indicated that despite a narrowing racial/ethnic “digital divide”, some disparities still exist, particularly among racial/ethnic groups with less education and whose primary language is not English. Findings will be used to inform development and evaluation of culturally tailored mHealth apps for use with interventions promoting healthy behavior change for Filipinos, Koreans, and Latinos.

## Introduction

Disparities exist among racial/ethnic populations, such as Latino and Asian American subgroups (eg, Filipinos and Koreans) who suffer from high rates of obesity, diabetes, and hypertension [1-5]. Healthy People 2020 recommends three key strategies for improving health outcomes and narrowing health disparities: (1) providing equitable access to health information, (2) improving health communications, and (3) providing quality health care [6].

Digital technology (mobile phones, tablets, and computers) holds great promise in helping reduce health disparities. It has been embraced as a means of facilitating health care services, such as providing electronic medical records, real-time health information, and remote delivery of preventive health interventions [7]. In particular, mobile phones and mobile health (mHealth) apps have the potential to overcome barriers to health care access, health information, and facilitate positive health outcomes [8,9]. For example, mobile phones can deliver lifestyle interventions promoting physical activity and health nutrition via mHealth apps that provide virtual health education, goal setting, social support, positive feedback, and coaching [10-12]. Moreover, mHealth apps that support self-monitoring of physical activity and dietary intake have been effective in promoting health behavior change and subsequent health outcomes (eg, weight loss) [13-15].

The theory of planned behavior supports the use of mHealth apps as a lifestyle intervention strategy. It posits that intent can be linked to personal motivation and is one of the most important predictors for behavior change [16]. Perceived control through self-monitoring of one’s behavior can motivate health behavior change, bolstering self-efficacy in one’s ability to succeed, thereby providing positive feedback to further improve health behaviors.

Encouraging evidence indicates the “digital divide” is narrowing between racial/ethnic minorities and the general population. Due to the proliferation, convenience, and affordability of digital technologies, racial/ethnic minorities have become avid mobile phone owners and users. Among the estimated 272 million US mobile phone users, penetration is highest for Asian Americans (78%) and Latinos (77%) [10,17].

Thus, coupling proven intervention strategies with mobile technologies, such as mobile phones and mHealth apps may be an effective and cost-effective means to promote healthy behaviors and improve health outcomes, particularly for at-risk racial/ethnic populations. Of the 17,000 commercially available mHealth apps, many are low cost or free, and offered in multiple languages (eg, Spanish) [13,18]. Approximately 30% of mobile phone users download at least one health app and of these health apps, 60% are related to diet and exercise [10,19].

However, a knowledge gap exists in the sparse datasets describing digital technology ownership and use among racial/ethnic subgroups, particularly information regarding the downloading of health apps for tracking health behaviors [9,10]. Asian Americans and Latinos are two of the largest and fastest growing US racial/ethnic minority populations [20]. Nonetheless, non-English speaking Asian Americans and Latinos were not specifically targeted in previous digital technology ownership and usage surveys [17,21]. Such information is important to facilitate the design and development of lifestyle interventions using mHealth apps to self-monitor health behaviors. This innovative strategy could promote healthy behavior change and thereby improve health outcomes for racial/ethnic populations at risk for lifestyle-related diseases.

Therefore, we conducted the Digital Link to Health (DiLH) study—a cross-sectional survey of diverse racial/ethnic groups, targeting Filipino, Korean, and Latino Americans at high risk for diabetes and cardiovascular disease. Results regarding gender difference in lay knowledge of type 2 diabetes are reported elsewhere [22]. The objective of this paper was to examine (1) types and extent of digital technology (mobile phones, computers, and tablets) ownership and usage (Internet access, social media, and health app), and (2) factors predicting the downloading of health apps among these populations. Results will help inform the development of culturally tailored mobile phone-delivered lifestyle interventions that use health apps—potentially improving health outcomes, and thus reducing health disparities for these at-risk racial/ethnic groups.

## Methods

### Study Design, Sample/Setting, Recruitment

The DiLH cross-sectional survey was conducted from August 2013 through December 2013 to develop a culturally tailored mobile phone-based diabetes prevention program for at-risk Filipinos, Koreans, and Latinos. Therefore, these specific racial/ethnic groups were over-sampled. The University of California, San Francisco, Institutional Review Board approved the study. A total of 1039 participants were recruited from the San Francisco Bay Area and San Diego community events, community clinics, churches, and online through Craigslist. A sample of 904 participants who were 18 years or older, self-identified as Caucasian, Filipino, Korean, or Latino, and with no history of diabetes were included in the final analysis. Excluded from analysis were individuals with missing data on gender (n=1), ethnicity/race (n=23), or identified as other Asian (n=53) or other race (n=58).

### Data Collection

The DiLH survey required 15 minutes to complete. The English DiLH survey was first pilot-tested among 10 participants. The survey was then forward and back translated into Spanish and Korean following published translation guidelines [23]. The English, Spanish, and Korean surveys incorporated revisions identified from the first pilot test and were pilot-tested a second time with 10 participants each.

Prior to administering the survey, bilingual staff screened potential participants. Surveys were administered either on paper or online (SurveyMonkey) and available in English, Spanish, or Korean. Participants independently completed the survey. Bilingual staff answered questions or assisted with the paper survey (ie, verbally administering the survey in preferred language). For those who preferred to complete the survey digitally, a link was available through Craigslist to the secure online SurveyMonkey site. Those who completed a paper survey were offered a complimentary tote bag. Those who completed the online survey had the option of entering a US $25 gift card raffle.

### Measures

The DiLH survey included questions regarding demographics, medical and family history, physical activity level, self-reported health status, and cardiovascular risk perceptions. Digital technology ownership was assessed by a “yes” or “no” answer to the question, “Do you currently own: a mobile phone (smartphone or non-smartphone), computer or laptop, and iPad or other type of tablet?” Digital technology usage (at least 1 time/week) was based on “yes” or “no” answers to questions pertaining to (1) mobile phone and/or computer usage, (2) Internet access via mobile phone and computer, (3) text messaging, and (4) email, Facebook, and/or Twitter usage. The primary outcome measure was determined by a “yes” or” no” answer to, “Do you have any health apps on your mobile phone or iPad/Tablet?” Participants were also asked to list any health apps they used. An investigator (SA) categorized and coded health apps as physical activity, diet/nutrition, health monitoring for blood pressure or blood glucose, or health information (eg, WebMD).

### Analysis

Data analysis was conducted using SPSS 22.0. Descriptive statistics characterized sociodemographics, digital technology ownership and usage for Caucasians, Filipinos, Koreans, and Latinos. After overall analyses determined that there was a significant difference in a categorical variable among the four race/ethnic groups, post hoc analyses were employed to determine between group differences for race/ethnicity, age, gender, marital status, education, years lived in the United States, language, survey type, and digital technology ownership and usage variables. One-way analysis of variance (ANOVA) was used to examine group differences in age. Chi-square analyses examined differences among the four racial/ethnic groups in the categorical variables. Binary logistic regression analyses were done to identify independent predictors associated with downloading a health app. A backward stepwise multiple logistic regression was run to determine the optimum combination of predictors associated with downloading a health app, where predictors remaining in the model showed significant unique contributions. Predictor variables considered for inclusion in the model were: race/ethnicity, age, gender, marital status, years lived in the United States, education, primary language is English, body mass index, smokes cigarettes, history of high blood pressure or high cholesterol, family member with diabetes, family member with myocardial infarction (MI), physical inactivity, perceived risk for MI, perceived risk for diabetes, self-reported health status, discussed diabetes with provider, and type of survey completed (online vs paper). These selected variables were added to the model because previous studies showed health behaviors differed based on these variables [5,24-28]. Statistical significance was set at *P*≤.05.

## Results

### Demographics

Table 1 provides demographic characteristics for 904 self-identified Caucasians, Latinos, Filipinos, and Koreans. Overall, mean age was 44 (SD 16.1) years. Koreans were the oldest (50, SD 14.3 years) and Filipinos were the youngest (41, SD 18.1 years). Overall, there were almost twice as many female (64.3%, 581/904) as male (35.7%, 323/904) participants. A total of 27.4% (247/904) of the participants had high school or less education. A majority of participants (87.5%, 788/904) were either native born or had lived ≥10 years in the United States. Among the racial/ethnic minority groups, Filipinos (67.6%, 169/250) were more likely to report English as their primary language compared to Latinos (17.7%, 44/248) and Koreans (9.4%, 22/234).

**Table 1:** Demographics and digital technology characteristics by race/ethnic groups.

Variable		All N=904	Caucasian n=172	Filipino n=250	Korean n=234	Latino n=248	Overall
		n (%)	n (%)	n (%)	n (%)	n (%)	*P* value
Age, years, mean (SD)		44 (16.1)	45 (16.1)	41 (18.1)	50 (14.3)	42 (14.0)	.001
**Gender**							.01
	Female	581 (64.3)	125 (72.7)	160 (64)	133 (56.8)	163 (65.7)	
	Male	323 (35.7)	47 (27.3)	90 (36)	101 (43.2)	85 (34.3)	
**Marital status** ^a^							<.001
	Married or cohabitating	524 (58.1)	68 (39.5)	117 (47.2)	189 (80.8)	150 (60.5)	
	Single or divorced	378 (41.9)	104 (60.5)	131 (52.8)	45 (19.2)	98 (39.5)	
**Education** ^a^							<.001
	High school or some high school	247 (27.4)	19 (11.0)	33 (13.3)	41 (17.5)	154 (62.6)	
	College or some college	515 (57.2)	109 (63.4)	187 (75.1)	136 (58.1)	83 (33.7)	
	Graduate school	139 (15.4)	44 (25.6)	29 (11.6)	57 (24.4)	9 (3.7)	
**Years lived in United States** ^a^							<.001
	<10 years	113 (12.5)	5 (2.9)	32 (12.8)	43 (18.5)	33 (13.4)	
	≥10 years	501 (55.6)	23 (13.4)	133 (53.2)	182 (78.1)	163 (66.3)	
	Native born	287 (31.9)	144 (83.7)	85 (34)	8 (3.4)	50 (20.3)	
Primary language English		404 (44.7)	169 (98.3)	169 (67.6)	22 (9.4)	44 (17.7)	<.001
**Type of survey**							<.001
	Online	250 (27.7)	105 (61)	49 (19.6)	58 (24.8)	38 (15.3)	
	Paper	654 (72.3)	67 (39.0)	201 (80.4)	176 (75.2)	210 (84.7)	

^a^Some variables have missing data, percentages are based on the n of each individual variable per group.

### Digital Technology Ownership

Overall, mobile phone ownership among all four groups (92.8%, 839/904) was almost twice that of landline phone ownership (54.8%, 165/904). Table 2 presents the adjusted digital technology ownership percentages by racial/ethnic groups, controlling for age, gender, marital status, education, years lived in the United States, language, and survey type. Landline ownership was highest among Filipinos (65.9%, 165/250) and lowest among Koreans (44.4%, 104/234). In contrast, mobile phone ownership was highest among Koreans (97.8%, 229/234) and lowest among Filipinos (89.0%, 223/250). However, smartphone ownership was highest among the Asian groups: Koreans (82.8%, 194/234) and Filipinos (81.7%, 204/250) compared to Caucasians (69.6%, 120/172) and Latinos (67.5%, 167/248).

### Digital Technology Usage

Among study participants, 96.1% (869/904) were mobile phone users. Internet access was higher via mobile phone (90.5%, 818/904) than computer (78.6%, 711/904). Table 2 also presents the adjusted digital technology usage percentages after controlling for demographic covariates. Mobile phone use was similar among all groups with no significant between group differences (overall *P=*.24). Regarding Internet access, Koreans were the most likely to access the Internet via mobile phone (95.7%, 224/234) and computer (96.7%, 226/234), compared to Caucasians (93.2%, 160/172 and 77.6%, 133/172) and Filipinos (88.2%, 221/250 and 78.7%, 197/250) respectively. Internet access was lowest for Latinos via mobile phone (85.4%, 212/248) and computer (61.0%, 151/248). Compared to all the other groups, Filipinos were the most likely to use: text messages (85.9%, 215/250), email (93.1%, 233/250), Facebook (87.4%, 219/250), and Twitter (48.0%, 120/250). Texting was similar between Caucasians (78.3%, 135/172), Latinos (79.8%, 198/248), and Koreans (79.7%, 186/234) (overall *P*<.093). Overall, groups preferred to use Facebook over Twitter by a ratio of 2 to 1.

Overall, 57.5% (520/904) of participants downloaded any type of app on their mobile phone or tablet. However, only 19.8% (179/904) of participants downloaded a health app. More Koreans (69.6%, 163/234) downloaded any type of app on their mobile phone or tablet compared to Caucasians (58.6%, 101/172), Filipinos (59.6%, 149/250), and Latinos (43.7%, 108/248) (overall *P*<.001). However, Koreans (17.8%, 42/234) and Latinos (12.1%, 30/248) were less likely than Caucasians (25.5%, 44/172) and Filipinos (24.7%, 62/250) to download health apps (overall *P*<.028). Notably, prevalence of any downloaded app and health app were similar for Caucasians (58.6%, 101/172 and 25.5%, 44/172, respectively) and Filipinos (59.6%, 149/250 and 24.7%, 62/250, respectively).

**Table 2:** Adjusted digital technology ownership and usage percentages by race/ethnic group.

Variable		All (N=904)	Caucasian (n=172)	Filipino (n=250)	Korean (n=234)	Latino (n=248)	Overall
		n (%)	n (%)	n (%)	n (%)	n (%)	*P* value^c^
**Digital technology ownership** ^d^							
	Landline phone	485 (54.8)	84 (49.3)	159 (65.9)	103 (44.4)	137 (57.2)	<.001
	Mobile phone^a^	825 (92.8)	160 (93.7)	215 (89)	228 (97.8)	222 (91.5)	.01
	Smartphone	622 (75.8)	115 (69.6)	181 (81.7)	183 (82.8)	144 (67.5)	<.001
	Computer/laptop	713 (80.8)	140 (81.8)	193 (80.4)	224 (96.6)	156 (65.3)	<.001
	iPad or tablet	340 (39.0)	60 (35.4)	98 (40.9)	124 (55.2)	59 (24.4)	<.001
**Digital technology usage** ^d^							
	Use mobile phone^a,b^	795 (96.1)	154 (92.7)	217 (96.7)	218 (98.2)	207 (96.1)	.24
	Use Internet via smartphone^b^	547 (90.5)	107 (93.2)	168 (88.2)	157 (95.7)	115 (85.4)	.02
	Use Internet via computer^b^	692 (78.6)	133 (77.6)	187 (78.7)	225 (96.7)	146 (61)	<.001
	Use text^b^	672 (81.2)	130 (78.3)	192 (85.9)	177 (79.7)	172 (79.8)	.09
	Use email^b^	683 (90.7)	145 (89.5)	198 (93.1)	199 (96.2)	141 (82.2)	.001
	Use Facebook^b^	456 (78.9)	112 (86.4)	156 (87.4)	87 (65.1)	101 (73.9)	.002
	Use Twitter^b^	100 (38)	22 (42.4)	42 (48)	25 (43.4)	12 (18)	.011
	Download any apps	509 (57.5)	100 (58.6)	144 (59.6)	161 (69.6)	106 (43.7)	<.001
	Download health apps	175 (19.8)	44 (25.5)	60 (24.7)	41 (17.8)	30 (12.2)	<.03

^a^Mobile phone=non-smartphones+smartphones.

^b^At least 1x/week in the last month.

^c^Adjusted for age, gender, marital status, education, years lived in the United States, language, survey type.

^d^Some variables have missing data, percentages are based on the n for each individual variable per group.

### Factors Predicting Health App Downloads

Table 3 presents results of a backward stepwise multiple logistic regression in predicting health app downloads controlling for demographic covariates. Only five out of the 18 variables remained in the final model. For each 1-year increase of age, the odds of downloading a health app were reduced by 4.0% (adjusted OR 0.96, 95% CI 0.95-0.97; *P*<.001). The odds of downloading a health app were reduced by 63% for Latinos (adjusted OR 0.37, 95% CI 0.20-0.69; *P*=.002) and 48% for Koreans (adjusted OR 0.52, 95% CI 0.31-0.88; *P*=.02) compared to Caucasians; and 50% for completing paper surveys (adjusted OR 0.50, 95% CI 0.34-0.75; *P*=.001) compared to online surveys. The odds of downloading a health app were 2.62 times greater for those who attended some or more college (adjusted OR 2.62, 95% CI 1.44-4.80; *P*=.002), and 2.93 times greater for those who attended graduate school (adjusted OR 2.93, 95% CI 1.43-6.00; *P*=.003) compared to those who had high school or less education. Those having a family member with a myocardial infarction (MI) had 2.02 times greater odds of downloading a health app (adjusted OR 2.02, 95% CI 1.16-3.51; *P*=.013) than those not having a family member with an MI.

### Popular mHealth Apps

Participants downloaded 187 health-related apps: 107 addressed diet, weight, and exercise, and 81 addressed health information. Participants could list more than one health app. The most popular downloaded mHealth apps were related to diet, weight, and exercise. The top downloaded mHealth apps were: The Calorie Counter, Lose It, and My Fitness Pal. The top downloaded health information apps were: Web MD and Kaiser Permanente apps.

**Table 3:** Multivariate logistic regression model for factors predicting the download of mobile health apps (N=848).^a^

Variable		Adjusted OR	95% CI	*P* value
Age (years)		0.96	0.95-0.97	<.001
**Race/ethnicity**				.002^b^
	Caucasian	reference		
	Filipino	0.89	0.54-1.48	.66
	Korean	0.52	0.31-0.88	.02
	Latino	0.37	0.20-0.69	.002
**Education**				.005^b^
	High school or some high school	reference		
	College or some college	2.62	1.44-4.80	.002
	Graduate school	2.93	1.43-6.0	.003
Family member with MI^c^		2.02	1.16-3.51	.013
**Type of survey**				.001
	Online survey	reference		
	Paper survey	0.50	0.34-0.75	

^a^Backward elimination step-wise multiple logistic regression (Wald). Variables entered in initial model: race/ethnicity, age, gender, marital status, years lived in the United States, education, primary language is English, body mass index, smokes cigarettes, has high blood pressure, has high cholesterol, family member with diabetes, family member with heart attack, physical inactivity, perceived risk for MI, self-reported health status, discussed diabetes with provider, and survey type completed.

^b^Overall *P* value.

^c^MI: myocardial infarction.

## Discussion

### Principal Findings

This study sought to examine digital technology ownership and usage among Caucasians, Filipinos, Koreans, and Latino Americans, and factors predicting health-seeking behaviors, specifically mobile phone health app downloads. Most large survey studies examining digital ownership and use focus on the general US English-speaking population. Moreover, Asian and Latino Americans subgroups are often characterized and analyzed as one aggregated group [4,29]. This survey study was unique in that it targeted specific racial/ethnic subgroups (Filipinos and Koreans) among whom less than half (44.7%, 404/904) considered English as their primary language.

Our findings support previous studies indicating that the “digital divide” between racial/ethnic groups is narrowing [8,30,31]. However, despite the increasing uptake of digital technology in the United States, we noted disparities still existed in our racial/ethnic groups, particularly among those who were older, had less education, and where English was not the primary language. In general, mobile phone ownership and Internet access was more equitable across groups, while online access and health-seeking behaviors (ie, health app downloads) demonstrated a wide variability.

### Digital Technology Ownership

The Pew Internet and American Life Project reported increasing computer and mobile phone ownership, with smartphone ownership increasing even faster [21]. While some Latino groups lag behind Caucasians in computer ownership (possibly due to affordability), mobile phone ownership is pretty much level among all racial/ethnic groups. Our overall mobile phone ownership (92.8%, 839/904) was similar to the Pew survey report of 91%. However, in comparison to the 58% reported by Pew survey for the general US population, all racial/ethnic groups in our study exhibited higher mobile phone ownership—with Koreans and Filipinos ranking highest, followed by Caucasians and Latinos. This supports the gradual demise of the digital divide as the convenience and affordability of digital technologies increase [30-33].

Evidence indicates that Asian Americans are the most avid owners and users of digital technology compared to all racial/ethnic groups [33]. In our study, Koreans were the most likely to own a mobile phone, computer, and/or iPad/tablet compared to Caucasians and Filipinos, while Latinos were the least likely to own these devices. Based on previous studies, higher education and income was correlated with digital technology ownership and usage, whereas age had an inverse correlation [8,9,34]. Koreans in our study were the most educated and oldest, while Latinos were the least educated and youngest, leading us to believe that education and income may have a stronger influence on digital ownership and use than age. Future research should examine additional factors contributing to ownership and use among race/ethnic subgroups.

### Digital Technology Usage

Internet access via mobile phones may be replacing computers due to a combination of convenience, functionality, and affordability, particularly among low-income populations [30,34]. The 2013 Pew Internet report found 63% of Americans access the Internet using mobile phones [35]. Moreover, one-third indicated their mobile phone is their primary Internet access, thus replacing computers as their primary access device. Similarly in our study, Internet access was higher via mobile phone than by computer among Caucasians, Filipinos, and Latinos. One exception was Koreans, who accessed the Internet equally by mobile phone and computer. Perhaps this is because Korean computer ownership was highest compared to all other groups. Nevertheless, mobile phone Internet access is increasing among multiple racial ethnic subgroups. Thus, digital technology tools, such as mHealth apps could empower individuals to take a more active role in their health care promoting health behavior change [9].

Social media survey reports indicate Asian Americans are the predominant online users of social networking sites worldwide [31,33]. For example, in a survey among the top 10 nations using Facebook, Filipinos (93.9%) were reported as the highest-ranked Facebook users [36]. In our study, Filipinos were the highest-ranked users of Facebook, Twitter, and text messaging. In contrast, Koreans were more likely to use email and text than other social media sites. This difference may be due to the inverse association of digital technology use with age [8,21,34]. Koreans on average were the oldest users of social media compared to Filipinos who were the youngest. In addition to this age bias, US digital technology use is skewed by native language, that is, most social media users are English dominant or bilingual [30]. The Pew Internet survey reported that the majority of Latinos (86%) who used social media sites were primarily English speakers. In contrast, only 17.7% (44/248) of Latinos in our study reported English as their primary language. However, a majority of the Latinos in our study reported using text, email, and Facebook (possibly in their native language), indicating an increased trend toward social media networking via mobile phone among non-English speaking Latinos, supporting a narrowing digital divide. Further research exploring the use of social media preferences among non-English speaking racial/ethnic groups is warranted.

Health information seeking behavior, such as downloading health apps is recognized as a vital activity in the “preparation stage” toward health behavior change [37]. National surveys found 75% of Americans use online sources for health information, illustrating health information is becoming more accessible, and health consumers are becoming more proactive [38,39]. Moreover, about 33% of Americans used mobile phones to access health information. Among mobile phone users, 20% downloaded at least one health app to track or manage their health. Reports indicated the most popular downloaded health apps addressed diet, weight, and exercise [11]. Our findings were similar wherein 19.8% (179/904) of participants downloaded at least one mobile health app. The top downloaded health apps in our study were for diet, weight, and exercise: The Calorie Counter, Lose It, and My Fitness Pal.

Factors predicting health app downloads included: race/ethnicity, age, education, family history of heart attack, and type of survey completed. Among the racial/ethnic subgroups, English-speaking and younger age groups (specifically Filipinos) were more likely to download mobile health apps than older and predominately native-speaking Koreans and Latinos. National surveys reported that individuals most likely to download health apps were younger individuals with higher education, while the least likely were Latinos [31,34]. Although language was not a predictor variable in our study, other studies found most US digital technology users were English dominant or bilingual, compared to non-English dominant users [9,28,30]. Since most US mobile health apps are in English, this may have deterred the predominately non-English speaking Latino and Korean groups from downloading health apps. Further research should assess potential psychographic drivers predicting the download of health apps, such as language preference, attitudes, and beliefs regarding culturally tailored health apps.

Having a family history of heart attack was also a predictor for downloading health apps. Participants with a family history of heart attack were two times more likely to download health apps than those without. Perceived risk for heart disease has been found to influence positive health behavior changes [25]. This may have motivated many of our study participants to download health-tracking apps. Curiously, a family history of diabetes was not a significant predictor for health app downloads, especially among Latinos and Filipinos who are at high risk [3,4]. Future research should explore this paradox, as well as examine mHealth apps as possible moderators for positive health behavior change, particularly among at-risk racial/ethnic populations.

Online survey participants in our study were younger by almost 6 years compared to paper survey participants. In general, previous findings indicated younger online survey participants were more likely to download health apps than older paper survey participants [21,34]. However, one study found older adults were more likely to seek health information than younger individuals [8]. A possible explanation is that younger individuals tend to be healthier than their older counterparts and thus, may be less likely to seek health information. It is also possible older participants search for health information on the Web, but do not download health apps as frequently as the younger, more tech savvy participants. However, further study is warranted to ascertain the reason for this age disparity.

### Strengths and Limitations

Several strengths and limitations for this study are noted. First, Asian Americans and Latinos are two of the largest and fastest growing US racial/ethnic minority populations, destined to be part of the majority by 2050 [20]. This survey study addresses the knowledge gap and adds to the body of science by way of datasets describing the digital technology ownership and use among specific Asian American subgroups (Filipinos and Koreans) and Latinos who have been largely overlooked in preventive health research. Moreover, our survey study was unique in that it targeted specific racial/ethnic subgroups whose primary language was not English. Second, the large sample size of diverse populations strengthens the validity of our findings that is representative of the US regions we sampled. However, our survey data was self-reported and the cross-sectional design prevents examination of causal relationships. Compared to national surveys, our study sample was comprised of specific racial/ethnic groups from the Western United States, with a younger age profile. Although the generalizability of our findings is limited, findings of the large diverse sample of Caucasians, Filipinos, Koreans, and Latinos from Northern and Southern California confirmed that a majority of these Latino and specific subgroup Asian populations were avid owners and users of digital technologies.

### Conclusions

Our study supports previous findings indicating the “digital divide” between racial/ethnic groups is narrowing. However, notable variability and some disparities still exist in digital technology ownership and usage reflecting pre-existing socioeconomic, cultural and difference in language proficiency. More importantly, results for this study add to the body of knowledge for digital technology ownership and use among specific Asian American subgroups and Latinos, including those whose primary language is not English.

Results indicated, although overall mobile phone ownership and usage in this California survey was similar among all groups, mobile phone ownership however, was significantly higher among participants than in national surveys. Digital technology ownership and usage varied by age and education, with younger and more educated individuals more likely to own and use digital technologies and download health apps.

In contrast to the more equitable distribution of digital technology ownership among our survey groups, the use of mobile health apps exhibited a wider variability and disparity, particularly in terms of age and race/ethnicity. Non-English speaking Latinos and Koreans groups were less likely to download health apps compared to predominantly English-speaking Caucasian and Filipinos. Moreover, downloading of health apps decreased with older age. Future research should explore factors influencing the use of digital technologies, specifically, health app use among racial/ethnic populations, particularly those with less English proficiency.

Finding from this study could be used to inform the design and development of culturally relevant self-monitoring mHealth apps for Filipinos, Koreans, and Latinos. Using health apps as a self-monitoring/tracking tool has the potential to promote health behavior change to improve health outcome and thereby mitigate health disparities among these at-risk populations. Recent studies evaluating lifestyle interventions using mobile technologies that allow for more proactive engagement through self-monitoring of health have found them to be effective in promoting healthy behavior change and disease management [40,41].

The narrowing digital divide between racial/ethnic populations is a first step. The next step is to develop culturally appropriate mHealth apps that are relevant, engaging, and tailored to today’s aging and diverse racial/ethnic health care consumer. Finally, the effectiveness of culturally appropriate mHealth apps to improve health outcomes for these at risk populations should be evaluated. This important research will provide evidence that mHealth apps could serve as innovative and interactive pathways to improve tomorrow’s health care outcomes and consumer well-being.

## Abbreviations

DiLH: Digital Link to Health
MI: myocardial infarction
